# Supplementary material for: The genomic landscape of metastatic castration-resistant prostate cancers reveals multiple distinct genotypes with potential clinical impact
Source: Nat Commun. 2019 Nov 20;10:5251. doi: 10.1038/s41467-019-13084-7 (PMC6868175; doi:10.1038/s41467-019-13084-7)
Supplement: Supplementary file 3 — Description of Additional Supplementary Files [file 41467_2019_13084_MOESM3_ESM.pdf]

Description of Additional Supplementary Files

File Name: Supplementary Data 1

Description: A - Clinical characteristics per mCRPC patient. Sheet B - Genomic characteristics per mCRPC patient. Sheet C -Overview of detected kataegis foci. Sheet D - Overview of kataegis foci characteristics. Sheet E - Overview of predicted fusions genes. Sheet F - Overview of predicted chromothripsis events. Sheet G - Sample categorization into unsupervised clusters (A-H). Sheet H - Mutually exclusive somatically mutated genes per clusters. Sheet I - Peaks detected by GISTIC2 and the type of CN aberration per sample. Sheet J - AR-enhancer and AR locus copy numbers per sample. Sheet K - dN/dS driver-gene discovery output based on somatic mutations. Sheet L - Mutational frequencies of TCGA-based clustering events. Sheet M - Comparison of mutational frequencies of top 200 mCRPC-mutated genes (dN/dS, GISTIC2, subtype-specific and supplemented with top mutated genes based on number of total aberrations) Sheet N - Comparison of mutational frequencies of top 200 mCRPC-mutated genes (dN/dS, GISTIC2, subtype-specific and supplemented with top mutated genes based on number of coding mutations)
