# Supplementary material for: The genomic landscape of metastatic castration-resistant prostate cancers reveals multiple distinct genotypes with potential clinical impact
Source: Nat Commun. 2019 Nov 20;10:5251. doi: 10.1038/s41467-019-13084-7 (PMC6868175; doi:10.1038/s41467-019-13084-7)
Supplement: Supplementary file 5 — Reporting Summary [file 41467_2019_13084_MOESM5_ESM.pdf]

nature research

Corresponding author(s): Martijn P. Lolkema

Last updated by author(s): Oct 4, 2019

Reporting Summary

Nature Research wishes to improve the reproducibility of the work that we publish. This form provides structure for consistency and transparency in reporting. For further information on Nature Research policies, see Authors & Referees and the Editorial Policy Checklist.

Statistics

For all statistical analyses, confirm that the following items are present in the figure legend, table legend, main text, or Methods section.

n/a	Confirmed	
☐	☒	The exact sample size (*n*) for each experimental group/condition, given as a discrete number and unit of measurement
☐	☒	A statement on whether measurements were taken from distinct samples or whether the same sample was measured repeatedly
☐	☒	The statistical test(s) used AND whether they are one- or two-sided<br>*Only common tests should be described solely by name; describe more complex techniques in the Methods section.*
☒	☐	A description of all covariates tested
☐	☒	A description of any assumptions or corrections, such as tests of normality and adjustment for multiple comparisons
☐	☒	A full description of the statistical parameters including central tendency (e.g. means) or other basic estimates (e.g. regression coefficient) AND variation (e.g. standard deviation) or associated estimates of uncertainty (e.g. confidence intervals)
☐	☒	For null hypothesis testing, the test statistic (e.g. *F*, *t*, *r*) with confidence intervals, effect sizes, degrees of freedom and *P* value noted<br>*Give P values as exact values whenever suitable.*
☒	☐	For Bayesian analysis, information on the choice of priors and Markov chain Monte Carlo settings
☒	☐	For hierarchical and complex designs, identification of the appropriate level for tests and full reporting of outcomes
☐	☒	Estimates of effect sizes (e.g. Cohen's *d*, Pearson's *r*), indicating how they were calculated

Our web collection on statistics for biologists contains articles on many of the points above.

Software and code

Policy information about availability of computer code

Data collection	WGS: HiSeq X Ten (V2.5 reagents), Illumina bcl2fastq tool (versions 2.17 to 2.20). BWA-mem v0.7.5a. GATK IndelRealigner and Haplotype Caller v3.4.46. GATK BQSR<br>ChIP-seq: Illumina HiSeq 2500, BWA, v0.5.10, MACS2, DFilter, MACS v1.4
Data analysis	All tools and scripts used for processing the WGS data are available at https://github.com/hartwigmedical/ and/or can be provided by authors upon request.<br>Open sources: Picard Tools v1.141 (CollectWgsMetrics), gnomAD database v2.0.2, TITAN v1.11.0, Strelka v1.0.12, Delly v0.5.5 and v0.7.8, R version 3.4.4. GISTIC2.0 v2.0.23, factoextra package v1.0.5, pvclust package v2.0, GenCode v28 (hg19), R package dndscv v0.0.0.9, tilingarray R package v1.56.0, R package karyoploteR v1.4.1, MutationalPatterns R package v1.4.2, NMF package v0.21.0, Shatterseek software v0.4, seriation package v1.2.3, prcomp R package

For manuscripts utilizing custom algorithms or software that are central to the research but not yet described in published literature, software must be made available to editors/reviewers. We strongly encourage code deposition in a community repository (e.g. GitHub). See the Nature Research guidelines for submitting code & software for further information.

Data

Policy information about availability of data

All manuscripts must include a data availability statement. This statement should provide the following information, where applicable:

- Accession codes, unique identifiers, or web links for publicly available datasets
- A list of figures that have associated raw data
- A description of any restrictions on data availability

WGS data and corresponding clinical data have been requested from Hartwig Medical Foundation and provided under data request number DR-011. The clinical data provided by CPCT have been locked at 1st of July 2018. Both WGS and clinical data is freely available for academic use from the Hartwig Medical Foundation through standardized procedures and request forms can be found at https://www.hartwigmedicalfoundation.nl.

Priestley, P. et al. Pan-cancer whole genome analyses of metastatic solid tumors. bioRxiv 415133 (2018). doi:10.1101/415133

Field-specific reporting

Please select the one below that is the best fit for your research. If you are not sure, read the appropriate sections before making your selection.

☒ Life sciences ☐ Behavioural & social sciences ☐ Ecological, evolutionary & environmental sciences

For a reference copy of the document with all sections, see nature.com/documents/nr-reporting-summary-flat.pdf

Life sciences study design

All studies must disclose on these points even when the disclosure is negative.

Sample size	Data from all available metastatic prostate cancer patients were requested, without performing sample-size calculation. To do any power calculation, one needs to have some established data from literature to estimate effects. To our knowledge this is the first large WGS analysis in metastatic prostate cancer. We deemed 197 metastatic lesions of metastatic patients sufficient for the analyses described.
Data exclusions	3 cases were excluded because biopsy was taken from primary tumor sample, 20 cases because they were not castrate-resistant prostate cancer subjects. 97 cases were excluded due to too low tumor percentage, 1 case due to too low DNA yield for WGS.
Replication	No replication was performed in obtaining WGS data; for each patient a biopsy and blood were sequenced once.
Randomization	Not relevant for this study, no prognostic or predictive analyses were performed.
Blinding	Not relevant for this study, no prognostic or predictive analyses were performed.

Reporting for specific materials, systems and methods

We require information from authors about some types of materials, experimental systems and methods used in many studies. Here, indicate whether each material, system or method listed is relevant to your study. If you are not sure if a list item applies to your research, read the appropriate section before selecting a response.

Materials & experimental systems

n/a	Involved in the study
☐	☒ Antibodies
☐	☒ Eukaryotic cell lines
☒	☐ Palaeontology
☒	☐ Animals and other organisms
☐	☒ Human research participants
☐	☒ Clinical data

Methods

n/a	Involved in the study
☐	☒ ChIP-seq
☒	☐ Flow cytometry
☒	☐ MRI-based neuroimaging

Antibodies

Antibodies used	Foxa1/2 (M-20, sc-6554 Santa Cruz Biotechnology), AR (N-20, sc-816 Santa Cruz Biotechnology), and H3K27ac (39133, Active Motif).
Validation	For antibody validation: we adhered to Encode criteria for antibody validation used for ChIP-seq analyses, in which an independent second antibody was used, specificity was confirmed using an independent secondary technology (immunofluorescence and westernblot), and further confirmation was performed as described in previous literature from our lab (AR: Stelloo 2015, 2018; FOXA1/2: Droog 2016; H3K27ac: Severson 2018, Droog 2016, 2017).

Eukaryotic cell lines

Policy information about cell lines

Cell line source(s)	VCaP and VCap-Bicalutamide resistant
Authentication	All cell lines (VCaP) were authenticated with Short Tandem Repeat (STR) profiling which supported correct heritage.
Mycoplasma contamination	VCaP cell lines were not tested for Mycoplasma contamination.

Commonly misidentified lines
(See ICLAC register)

Name any commonly misidentified cell lines used in the study and provide a rationale for their use.

Human research participants

Policy information about studies involving human research participants

Population characteristics	Patients of ≥ 18 years with metastatic castrate-resistant prostate cancer of whom a histological biopsy could be safely obtained and for whom systemic treatment with anti-cancer agents was indicated, were eligible for inclusion. All patients gave written informed consent prior to any study procedure.
Recruitment	Patients with metastatic prostate cancer who were included under the protocol of the Centre for Personalized Cancer Treatment (CPCT) consortium (NCT01855477).
Ethics oversight	This CPCT-02 protocol was approved by the medical ethical committee (METC) of the University Medical Center Utrecht.

Note that full information on the approval of the study protocol must also be provided in the manuscript.

Clinical data

Policy information about clinical studies

All manuscripts should comply with the ICMJE guidelines for publication of clinical research and a completed CONSORT checklist must be included with all submissions.

Clinical trial registration	NCT01855477
Study protocol	https://clinicaltrials.gov/ct2/show/NCT01855477
Data collection	Patients were included for biopsy between 03 May 2016 and 28 May 2018. Clinical data, including tumor characteristics, previous therapies and therapy and outcome after study inclusion, were collected in electronic case record forms and stored in a central database.
Outcomes	Primary Outcome Measure: Percentage of patients enrolled in clinical intervention trials based on the mutational profile of their cancer genome<br><br>Secondary Outcome Measures :<br>- Percentage of samples with sufficient DNA for sequencing analysis<br>- Percentage of samples with an adequate mutational profile to allow enrollment in trials<br>- Differences in mutational profile pre, post and during treatment<br>- Number and nature of (serious) adverse events of the performed histological biopsies

ChIP-seq

Data deposition

☒ Confirm that both raw and final processed data have been deposited in a public database such as GEO.

☒ Confirm that you have deposited or provided access to graph files (e.g. BED files) for the called peaks.

Data access links<br>*May remain private before publication.*	https://www.ncbi.nlm.nih.gov/geo/query/acc.cgi?acc=GSE138168
Files in database submission	AR_LNCAP_with_R1881_MACS2Peaks.bed<br>AR_LNCAP_with_R1881.bam.coverage.bedgraph<br>AR_LNCAP_without_R1881_MACS2Peaks.bed<br>AR_LNCAP_without_R1881.bam.coverage.bedgraph<br>AR_Patient1_MACS2Peaks.bed<br>AR_Patient1.bam.coverage.bedgraph<br>AR_Patient2_MACS2Peaks.bed<br>AR_Patient2.bam.coverage.bedgraph<br>AR_PatientA_PRAD_MACS2Peaks.bed<br>AR_PatientA_PRAD_Reads.bam.coverage.bedgraph<br>AR_PatientB_PRAD_MACS2Peaks.bed<br>AR_PatientB_PRAD_Reads.bam.coverage.bedgraph<br>AR_PatientC_PRAD_MACS2Peaks.bed<br>AR_PatientC_PRAD_Reads.bam.coverage.bedgraph<br>AR_VCAP_bicalutamide_resist_MACS2Peaks.bed<br>AR_VCAP_bicalutamide_resist.bam.coverage.bedgraph<br>AR_VCAP_MACS2Peaks.bed<br>AR_VCAP.bam.coverage.bedgraph<br>FOXA1_LNCAP_with_R1881_MACS2Peaks.bed<br>FOXA1_LNCAP_with_R1881.bam.coverage.bedgraph<br>FOXA1_Patient1_MACS2Peaks.bed

FOXA1_Patient1.bam.coverage.bedgraph
FOXA1_Patient2_MACS2Peaks.bed
FOXA1_Patient2.bam.coverage.bedgraph
FOXA1_PatientA_PRAD_MACS2Peaks.bed
FOXA1_PatientA_PRAD_Reads.bam.coverage.bedgraph
FOXA1_PatientB_PRAD_MACS2Peaks.bed
FOXA1_PatientB_PRAD_Reads.bam.coverage.bedgraph
FOXA1_PatientC_PRAD_MACS2Peaks.bed
FOXA1_PatientC_PRAD_Reads.bam.coverage.bedgraph
H3K27AC_LNCAP_DSGfixing_MACS2Peaks.bed
H3K27AC_LNCAP_DSGfixing.bam.coverage.bedgraph
H3K27ac_Patient1_MACS2Peaks.bed
H3K27AC_Patient1.bam.coverage.bedgraph
H3K27ac_Patient2_MACS2Peaks.bed
H3K27AC_Patient2.bam.coverage.bedgraph
H3K27ac_PatientA_PRAD_MACS2Peaks.bed
H3K27ac_PatientA_PRAD_Reads.bam.coverage.bedgraph
H3K27ac_PatientB_PRAD_MACS2Peaks.bed
H3K27ac_PatientB_PRAD_Reads.bam.coverage.bedgraph
H3K27ac_PatientC_PRAD_MACS2Peaks.bed
H3K27ac_PatientC_PRAD_Reads.bam.coverage.bedgraph

Genome browser session
(e.g. UCSC)

Provide a link to an anonymized genome browser session for "Initial submission" and "Revised version" documents only, to enable peer review. Write "no longer applicable" for "Final submission" documents.

Methodology

Replicates

Patient samples from 2 individuals are used as a biological replicate.

Sequencing depth

Immunoprecipitated DNA was processed for sequencing using standard protocols and sequenced on an Illumina HiSeq 2500 with 65bp single end reads.

Antibodies

AR (N-20, sc-816 Santa Cruz Biotechnology); Foxa1/2 (M-20, sc-6554 Santa Cruz Biotechnology); H3K27ac (39133, Active Motif)

Peak calling parameters

For all the patient samples:
-Macs2 command line:
macs2 callpeak -t sample.bam -c control.bam -f BAM --gsize hs -n sample --outdir output/ -q 0.01 --extsize=$fragment –nomodel
where $fragment= estimated fragment size for the sample.bam from phantompeaks

-DFIlter command line:
run_dfilter.sh -d=sample.bed -c=input.bed -o=sample.dfilter -bs=50 -ks=30 -refine

-phantompeaks command line:
run_spp.R -rf -c=sample.bam -i=input.bam -savp

-bedtools intersectBed command on the macs2 and dfilter results:
bedtools intersect -a sample.macs2 -b sample.dfilter > sample.peak.intersect

Data quality

For all patient samples, all peaks are below FDR 5% and at least 80% peaks are above 5-fold enrichment .

Software

macs2 version 2.1.1.20160309; DFilter version 1.5; phantompeakqualtools version spp_1.14.tar.gz
